# Supplementary material for: Sex-Specific Differences in Toxicity Following Systemic Paclitaxel Treatment and Localized Cardiac Radiotherapy
Source: Cancers (Basel). 2021 Aug 6;13(16):3973. doi: 10.3390/cancers13163973 (PMC8394799; doi:10.3390/cancers13163973)
Supplement: Supplementary file 1 [file cancers-13-03973-s001.zip › cancers-1289473-supplementary.pdf]

cancers

MDPI

Supplementary Material: Sex-Specific Differences in Toxicity Following Systemic Paclitaxel Treatment and Localized Cardiac Radiotherapy

Nicole Chmielewski Stivers, Benoit Petit, Jonathan Ollivier, Virginie Monceau, Pelagia Tsoutsou, Ana Quintela Pousa, Xiaoming Lin, Charles Limoli and Marie-Catherine Vozenin

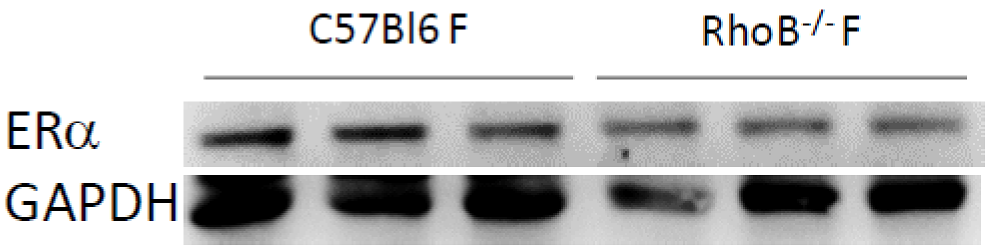

Figure S1: Western-blot analysis of ERα. The analysis of ERα expression level performed in heart lysate of WT and *rhoB* deficient females showed lower level of ERα in *rhoB* deficient animals. GAPDH was used as loading control.

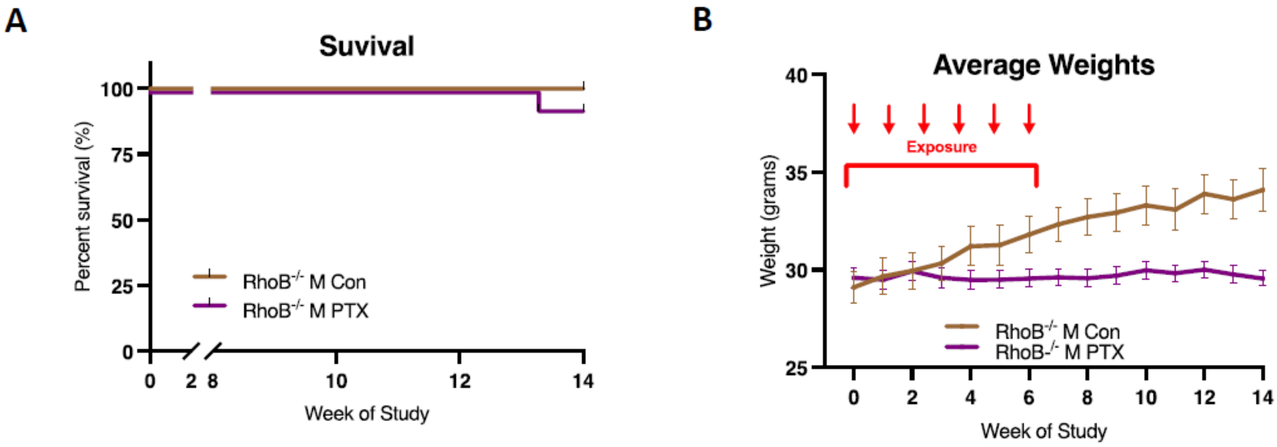

Figure S2: *RhoB* deficiency in males. **A)** After 150 mg/kg paclitaxel treatment, survival analysis shows that 1 male died in *rhoB* deficient males (n = 14) vs controls (n = 10). **B)** *RhoB* deficient males showed no weight up-take as compared with untreated males. 2-way ANOVA analysis demonstrated significant interaction (p = 0.0022) and treatment (< 0.0001) effects. Chart uses mean ± s.e.m.
